# S-Layer Glycoprotein From *Lactobacillus kefiri* Exerts Its Immunostimulatory Activity Through Glycan Recognition by Mincle

**DOI:** 10.3389/fimmu.2019.01422

**Published:** 2019-06-26

**Authors:** Mariano Malamud, Paula Carasi, Matías H. Assandri, Teresa Freire, Bernd Lepenies, María de los Ángeles Serradell

**Affiliations:** ^1^Cátedra de Microbiología, Departamento de Ciencias Biológicas, Facultad de Ciencias Exactas, Universidad Nacional de La Plata, La Plata, Argentina; ^2^Immunology Unit & Research Center for Emerging Infections and Zoonoses (RIZ), University of Veterinary Medicine Hannover, Hannover, Germany; ^3^CCT La Plata, CONICET, Instituto de Estudios Inmunológicos y Fisiopatológicos (IIFP), La Plata, Argentina; ^4^Laboratorio de Inmunomodulación y Desarrollo de Vacunas, Departamento de Inmunobiología, Facultad de Medicina, Universidad de la República, Montevideo, Uruguay; ^5^Instituto de Ciencias de la Salud, Universidad Arturo Jauretche, Florencio Varela, Argentina

**Keywords:** S-layer protein, adjuvants, C-type lectin receptors, DCs activation, lactobacillus

## Abstract

The development of new subunit vaccines has promoted the rational design of adjuvants able to induce a strong T-cell activation by targeting specific immune receptors. The S-layer is a (glyco)-proteinaceous envelope constituted by subunits that self-assemble to form a two-dimensional lattice that covers the surface of different species of *Bacteria* and *Archaea*. Due to their ability to self-assemble in solution, they are attractive tools to be used as antigen/hapten carriers or adjuvants. Recently, we have demonstrated that S-layer glycoprotein from *Lactobacillus kefiri* CIDCA 8348 (SLP-8348) enhanced the LPS-induced response on macrophages in a Ca^2+^-dependent manner, but the receptors involved in these immunomodulatory properties remain unknown. Therefore, we aim to determine the C-type lectin receptors (CLRs) recognizing this bacterial surface glycoprotein as well as to investigate the role of glycans in both the immunogenicity and adjuvant capacity of SLP-8348. Here, using a mild periodate oxidation protocol, we showed that loss of SLP-8348 glycan integrity impairs the cell-mediated immune response against the protein. Moreover, our data indicate that the adjuvant capacity of SLP-8348 is also dependent of the biological activity of the SLP-8348 glycans. In order to evaluate the CLRs involved in the interaction with SLP-8348 an ELISA-based method using CLR–hFc fusion proteins showed that SLP-8348 interacts with different CLRs such as Mincle, SingR3, and hDC-SIGN. Using BMDCs derived from CLR-deficient mice, we show that SLP-8348 uptake is dependent of Mincle. Furthermore, we demonstrate that the SLP-8348-induced activation of BMDCs as well as its adjuvant capacity relies on the presence of Mincle and its signaling adaptor CARD9 on BMDCs, since SLP-8348-activated BMDCs from Mincle^−/−^ or CARD9^−/−^ mice were not capable to enhance OVA-specific response in CD4^+^ T cells purified from OT-II mice. These findings significantly contribute to the understanding of the role of glycans in the immunomodulation elicited by bacterial SLPs and generate a great opportunity in the search for new adjuvants derived from non-pathogenic microorganisms.

## Introduction

Studies of the biotechnological applications of S-layer proteins, considered as one of the most abundant biopolymers on our planet (1), have been increasing in recent years. The S-layer is a surface macromolecular array, found on both Gram-positive and Gram-negative bacteria and highly prevalent in archaea (2). It is generally formed by identical protein or glycoprotein subunits that are held together by non-covalent interactions and self-assemble to form a two-dimensional lattice (3, 4). Since the S-layer constitutes the outermost surface structure in those microorganisms, it is in direct contact with the bacterial environment and could mediate interactions with host cells. This feature, together with the ability to self-assemble and its unique physicochemical properties, make S-layer proteins (SLPs) an attractive tool to use as antigen/hapten carrier, as adjuvant, or as part of vaccination vesicles (1). Therefore, understanding how SLPs interact with immune cells is a critical step toward the application of these proteins in the development of new adjuvanted vaccines.

It is known that upon recognition of microbial structures through different pattern recognition receptors (PRRs), antigen presenting cells (APCs) such as dendritic cells (DCs) and macrophages, undergo signal transduction that lead to cell maturation with the up-regulation of co-stimulatory molecules and production of different chemokines and cytokines. There are different classes of PRR families, including transmembrane proteins such as Toll-like receptors (TLRs) and C-type lectin receptors (CLRs) (5). TLRs are a family of membrane-bound proteins that mainly recognize microbial membrane components such as lipids, lipoproteins, and proteins (6). In contrast, CLRs are specialized in the recognition of carbohydrates, through one or more carbohydrate recognition domains (CRDs). Depending on the receptors engaged, APCs display different maturation states and produce different inflammatory mediators that impact the following cellular and humoral responses (7, 8).

*Lactobacillus kefiri* is a lactic acid bacterium derived from kefir, carrying an S-layer glycoprotein in its envelope (9–11). It has been shown that *L. kefiri* has immunomodulatory properties, and both *in vitro* and *in vivo* experiments support its potential as a probiotic microorganism (12, 13). Moreover, the SLPs from *L. kefiri* antagonize the effect of *Clostridium difficile* toxins on Vero cells (14), mediate the inhibition of *Salmonella enterica* invasion to Caco-2 cells (15) and they also enhance the adhesion of *L. kefiri* to gastrointestinal mucus (16).

Glycosylation is the most frequent post-translational modification found on SLPs (17). Some studies have been conducted to address the role of the glycans present in the SLPs from different *Lactobacillus* species in their immunomodulatory properties. In LPS-treated immature dendritic cells, the SLP from *L. acidophilus* NCFM was able to induce an anti-inflammatory profile, mediated by the CLR DC-specific ICAM-3-grabbing non-integrin (DC-SIGN) (18). Moreover, this SLP-DC-SIGN engagement was demonstrated for S-layer like proteins from *L. plantarum* (19) and was also suggested for the SLP from *L. kefiri* JCM 5818 (20).

Recently, we have demonstrated that the S-layer glycoprotein from *Lactobacillus kefiri* CIDCA 8348 (SLP-8348) was internalized by macrophages in a process that was mediated by carbohydrate-receptor interactions. In addition, SLP-8348 enhanced the LPS-induced response on macrophages in a Ca^+2^-dependent manner (21). However, the molecular mechanisms as well as the receptors involved in the immunomodulatory properties elicited by SLP-8348 on APCs are not well-understood. Thus, the aim of this work was to investigate the involvement of CLRs in the immune cell response to SLP-8348 using *in vitro* and *in vivo* approaches.

## Materials and Methods

### Bacterial Strains and Growth Conditions

*L. kefiri* CIDCA 8348 isolated from kefir grains was used (22). The strain was cultured in de Man-Rogosa-Sharpe (MRS) broth (Difco, Beauvais, France) at 37°C for 48 h in aerobic conditions. Frozen stock cultures were stored at −80°C in skim milk until use.

### S-Layer Protein Extraction

S-layer protein extraction from bacterial cells at stationary phase was performed using 5 M LiCl as previously described (14). SLPs extracts were tested by sodium dodecylsulphate-polyacrylamide gel electrophoresis (SDS-PAGE) in 12% separating and 4% stacking gels using the discontinuous buffer system according to Laemmli (23). Gels were migrated on a BioRad Mini-Protean II (BioRad Laboratories, Richmond, CA, USA) and revealed using Colloidal Blue Staining. Carbohydrate glycol groups present in SLP-8348 were oxidized with sodium periodate (10 mM) as previously described by Rodriguez et al (24). The oxidation was performed at room temperature for 45 min in the dark, followed by the reduction with sodium borohydride (50 mM) of the reactive aldehyde groups (24). The resulting oxidized SLP is referred as SLPOx-8348. The control, SLPB-8348, consisted of SLP-8348 subjected to the whole treatment excepting for the incubation with sodium periodate. SLPs were dialyzed against PBS and then filtrated through a membrane of 0.45 μm pore diameter. Protein concentration was determined according to Bradford (25).

### Cell Cultures

The monocyte/macrophage murine cell line RAW 264.7 was cultured in Dulbecco's Modified Eagle Medium (DMEM) supplemented with: 10% (v/v) heat-inactivated (30 min/60°C) fetal bovine serum (FBS), 1% (v/v) non-essential amino acids and 1% (v/v) penicillin-streptomycin solution (100 U/mL penicillin G,100 g/mL streptomycin). All cell culture reagents were from GIBCO BRL Life Technologies (Rockville, MD, USA).

BMDCs were generated from C57BL/6 wild type, Mincle ^−/−^, CARD9 ^−/−^, or SignR3 ^−/−^ bone marrow precursors (2.5 × 10^5^ cells/ml) that were plated in complete culture medium (IMDM supplemented with 2 mM glutamine, 10% (v/v) FBS, 100 U/ml penicillin and 100 μg/ml streptomycin) supplemented with a GM-CSF containing supernatant from P3-X63 cells. Medium was exchanged every 48 h and BMDCs were used after 8–10 days of differentiation to ascertain that ≥80% of the cell population expressed the marker CD11c.

### Binding and Internalization of SLP-8348 by BMDCs

Binding and internalization of SLP-8348 were analyzed by flow cytometry. Labeling of SLP-8348 with Atto 647 N was performed according to the manufacturer's instructions (SIGMA, USA). BMDCs from C57BL/6 mice, Mincle-deficient and SignR3-deficient mice (2.5 × 10^5^/mL) were incubated with Atto 647 N-labeled SLP-8348 for 1 h at 37°C (to assess uptake), or at 4°C in complete medium (to assess binding) (21, 24). Cells were then washed, incubated with mouse anti-CD11c antibody and analyzed by flow cytometry.

### Cells Stimulation Assays

RAW 264.7 cells (2.5 × 10^5^) were distributed onto 24-well microplates (JET BIOFIL®, China), and the medium volume was adjusted to 0.5 mL. The plates were incubated for 48 h at 37°C in a 5% CO_2_ 95% air atmosphere to allow cell adherence prior to experimentation. After that, cells were treated with SLP-8348 (10 μg/mL), SLPOx-8348 (10 μg/mL) or SLPB-8348 (10 μg/mL) in the presence or absence of LPS 0.1 μg/mL (LPS from *Escherichia coli* O111:B4, SIGMA, USA), in DMEM for 24 h at 37°C in a 5% CO_2_ 95% air atmosphere. Cells incubated with DMEM were used as negative control.

BMDCs from wild type or CLR-deficient mice (1 × 10^5^ cells/well) were distributed onto 96-well microplates (Sarstedt, Germany) and stimulated with SLP-8348 (10 μg/mL), LPS (0.25 μg/mL) or the combination of both for 16 h at 37°C in a 5% CO_2_ 95% air atmosphere. Culture supernatants were collected and analyzed by ELISA for IL-6 and TNF-α secretion. Cells were incubated with anti-CD16/32 (93) to block cell surface FcγRII/RIII receptors, stained with anti-CD11c (N418), CD40 (3/23), CD80 (16-10A1) and then analyzed by flow cytometry.

### Mice

For *in vivo* experiments, 6- to 8-week-old female BALB/c mice were purchased from the Animal Care Facility of the Facultad de Ciencias Veterinarias of the Universidad Nacional de La Plata (Argentina) or DILAVE Laboratories (Uruguay). Animals were kept in the animal house (Cátedra de Microbiología, Facultad de Ciencias Exactas, UNLP, La Plata, Argentina or URBE, Facultad de Medicina, UdelaR, Montevideo, Uruguay) with water and food supplied *ad libitum*, and handled in accordance with institutional guidelines for animal welfare.

C57BL/6, C57BL/6-Tg (TcraTcrb)425Cbn/J mice (OT-II transgenic mice), Mincle ^−/−^, CARD9 ^−/−^ or SignR3 ^−/−^ were kept in the animal house of the University of Veterinary Medicine (Hannover, Germany) with water and food supplied *ad libitum*. Mice were sacrificed for the isolation of spleen cells (OT-II transgenic mice) or the preparation of bone marrow for BMDC generation (Mincle^−/−^, CARD9^−/−^, SignR3^−/−^, and WT mice).

### BMDC/T Cell Co-culture Assay

Splenocytes were isolated from OT-II transgenic mice by flushing the spleen with complete IMDM medium. After erythrocyte lysis, cells were resuspended in MACS buffer (0.5% (w/v) BSA, 2 mM EDTA in PBS). T cells were obtained by magnetic-activated cell separation (MACS) using Pan T cell isolation kit II, mouse (Miltenyi Biotech, Bergisch Gladbach, Germany) according to the manufacturer's instructions. T cells were labeled with CFSE (carboxyfluorescein diacetate succinimidyl ester, Sigma-Aldrich, USA) and seeded on a 96-well round bottom culture plate (7 × 10^4^ cells/well). After 30 min, BMDCs treated with OVA (Endo Grade® Ovalbumin, LIONEX GmbH, Germany) or OVA/SLP-8348 were added and incubated for 72 h. Culture supernatants were collected for IFN-γ secretion.

### Immunization

To test the SLP-8348 immunogenicity, BALB/c mice (8 weeks old, 5 mice per group) were subcutaneously injected on the base of the tail with one dose of PBS, SLP-8348 (10 μg/mouse), SLPOx-8348 (10 μg/mouse), SLPB-8348 (10 μg/mouse), OVA (10 μg/mouse) or the combinations of them, with or without IFA. After 10 days cells from inguinal lymph nodes were labeled with CFSE and stimulated *in vitro* with SLP-8348 (10 μg/ml) or OVA (10 μg/ml) for 5 days. Experimental protocol was approved by the Institutional Animal Care and Use Committee of the Facultad de Ciencias Exactas, Universidad Nacional de La Plata (Protocol 006-00-18).

### C-Type Lectin Receptor Recognition of S-Layer Protein

The CLR-reactivity on SLP-8348 was evaluated by ELISA (26). A half-area microplate (Greiner Bio-One GmbH, Frickenhausen, Germany) was coated with 0.25 μg SLP-8348, SLPOx-8348, or SLPB-8348 per well for 16 h at 4°C and blocked with 1% (w/v) BSA (Thermo Fisher Scientific, Darmstadt, Germany) for 2 h at room temperature (RT). Then, 0.25 μg of each CLR-hFc fusion protein in lectin-binding buffer (50 mM HEPES, 5 mM MgCl_2_, and 5 mM CaCl_2_) was added and incubated for 1 h at RT. For inhibition assays, CLR-hFc fusion proteins were pre-incubated with 5 mM of EGTA (Sigma-Aldrich, USA). After washing, HRP-conjugated anti-human IgG antibody (Dianova) was added to each well for 1 h at RT. Finally, plates were incubated with chromogenic substrate (o-phenylenediamine dihydrochloride substrate tablet (Thermo Fisher Scientific), 24 mM citrate buffer, 0.04% (v/v) H_2_O_2_, 50 mM phosphate buffer in H_2_O). The reaction was stopped with 2.0 M sulfuric acid and the plate was read at 495 nm using a Multiskan Go microplate spectrophotometer (Thermo Fisher Scientific).

### Cytokine Quantification in Cell Culture Supernatants

Production of IL-6 by macrophages and IFN-γ by cells from inguinal lymph nodes were analyzed by sandwich ELISA using commercially available capture and detection antibodies from BD-Pharmingen (San Diego, USA). Secretion of IL-6 and TNF-α by BMDCs and IFN-γ from purified T cells were analyzed by ELISA from PeproTech (USA). The assays were performed according to the manufacturer's instructions. After determining optical densities, cytokine levels in cell culture supernatants were calculated using the GraphPad Prism 6.0 program.

### Immunocytostaining and Flow Cytometry

After stimulation experiments, cells were washed twice with PBS containing 2% (v/v) FBS and then labeled with specific antibodies for 30 min at 4°C. Cells were washed twice with PBS containing 2% (v/v) FBS, and then fixed with 1% (v/v) formaldehyde. Cells were analyzed using a FACSCalibur Analyzer (BD Biosciences), Accuri C6 (BD Biosciences) or Attune NxT Flow Cytometer (Thermo Fisher Scientific). Data analysis was performed using the FlowJo Software (FlowJo, Ashland, OR, USA).

### Statistical Analysis

Statistical analysis was performed with the GraphPad Prism program. Values from at least three independent experiments were analyzed by using a one-way or two-way ANOVA with Tukey's *post hoc* test (*p* < 0.05 was considered statistically significant).

## Results

### SLP-8348 Glycan Moieties Modulate the TLR-Induced Maturation of Macrophages

**Figure 1:**
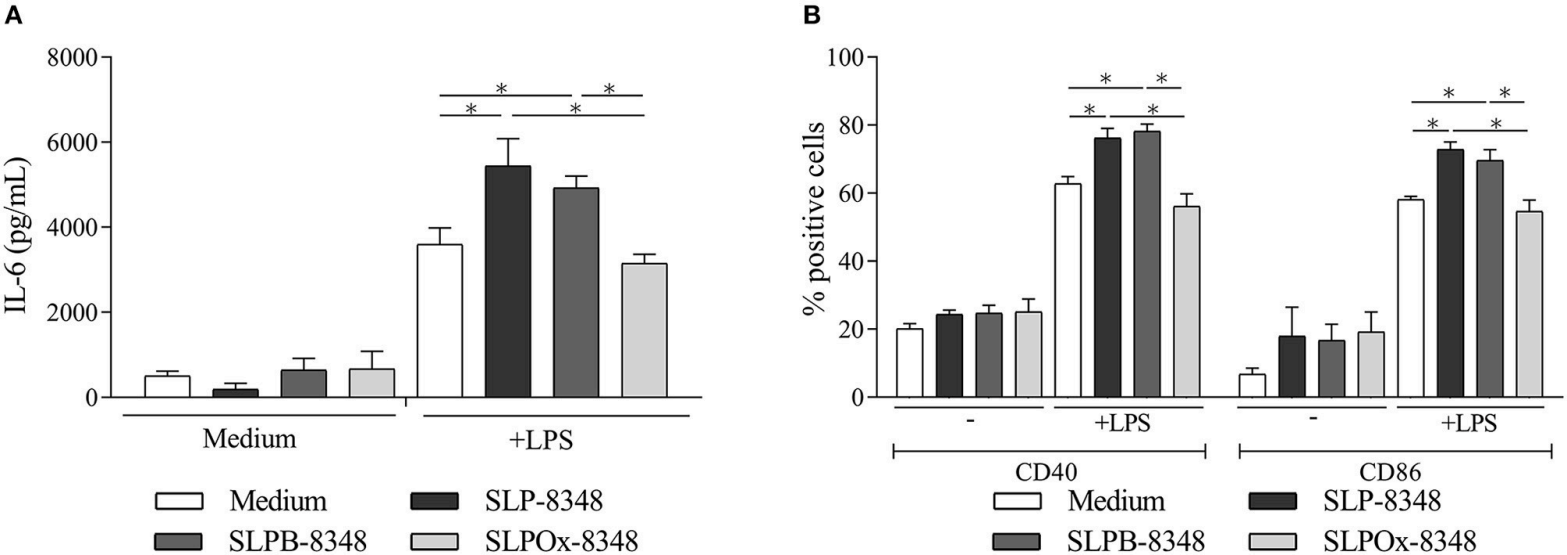
Glycans from SLP-8348 modulate TLR-induced maturation of macrophages. IL-6 concentrations (pg/mL) by capture ELISA in the supernatant of the murine RAW 264.7 cultures after 24 h of stimulation **(A)**. Percentage of CD40^+^ and CD86^+^ RAW 264.7 cells after 24 h of stimulation **(B)**. A representative figure of three independent experiments is shown (±SD, indicated by error bars). Asterisks indicate statistically significant differences by one-way ANOVA with Tukey's *post hoc* test (^*^p < 0.05).

Previous studies performed in our laboratory demonstrated the capacity of SLP-8348 to enhance *in vitro* TLR-induced maturation of murine macrophages in a Ca^+2^-dependent manner (21). In order to determine if the carbohydrates from SLP-8348 participate in the induction of high levels of macrophage activation, we performed a mild periodate oxidation of glycans where the glycol groups in carbohydrates are oxidized to reactive aldehyde groups, which are in turn reduced with sodium borohydride. During this process, the glycans and the integrity of glycoproteins are maintained, but instead the molecular conformation of the glycans is altered, losing possible biological activity (24). Macrophages stimulated with oxidized S-layer protein (SLPOx-8348) and LPS produced similar levels of IL-6 as well as a comparable expression of co-stimulatory molecules CD40 and CD86 as cells incubated with LPS alone (Figure 1). Macrophages incubated with the control SLPB-8348 and LPS, developed the same stimulation profile as cells in presence of LPS and SLP-8348, with higher levels of IL-6, CD40 and CD86 compared to LPS-treated macrophages (Figure 1). These results indicate that terminal glycans present on SLP-8348 determine the immunostimulatory activity on LPS-matured macrophages.

### Glycan Oxidation Impairs the Immunogenicity of SLP-8348

**Figure 2:**
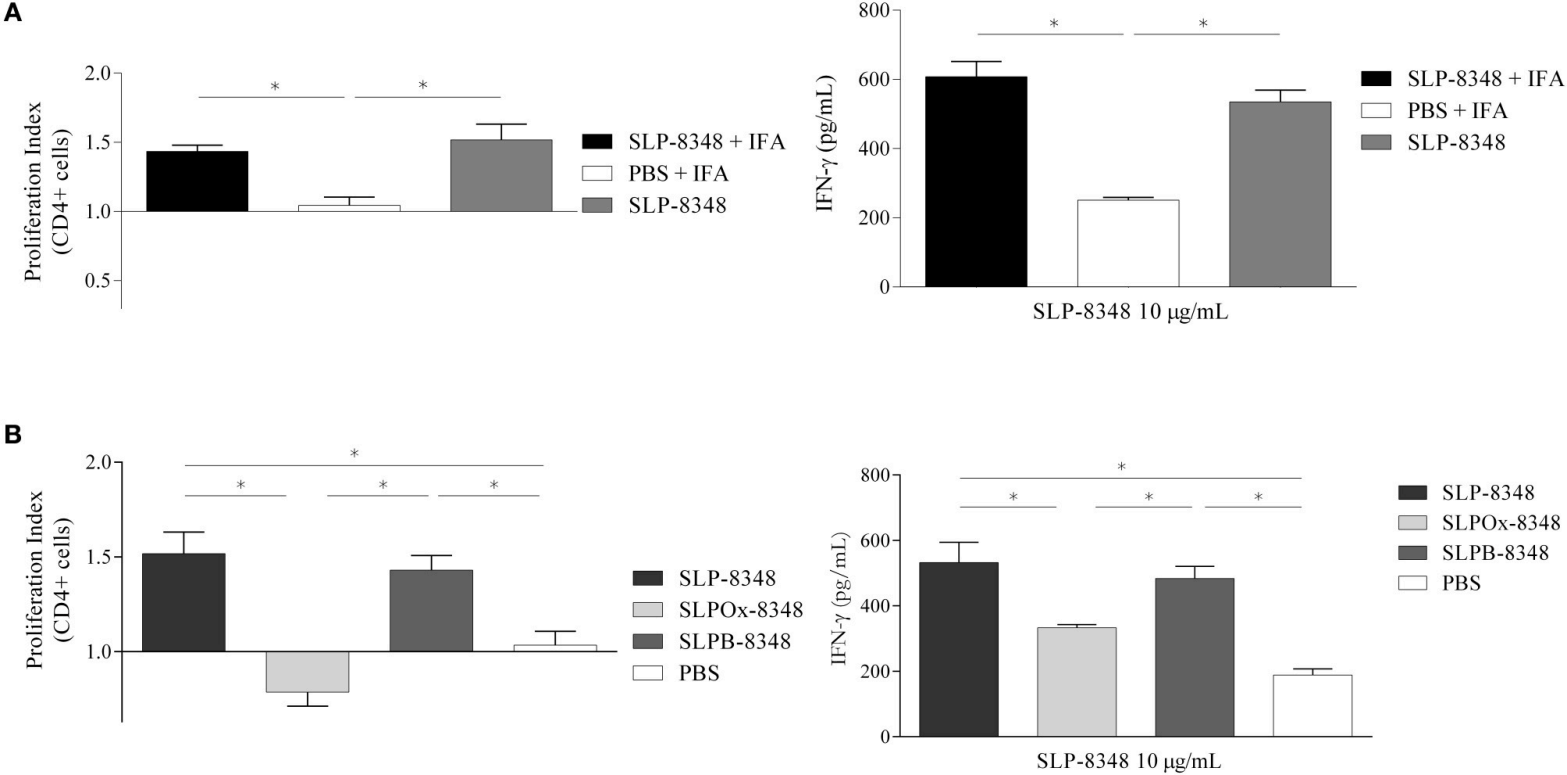
Glycan residues are crucial in SLP-8348 immunogenicity. Cells from inguinal lymph nodes were obtained from BALB/c mice subcutaneously injected with one dose (10 μg/mouse) of SLP-8348 or PBS in combination or not with incomplete Freund's adjuvant. IFN-γ levels on supernatants and proliferation index of CD4^+^ T cells (calculated as the ratio between the percentage of CFSE ^low^ CD4^+^ cells and CFSE^low^ CD4^+^ cells from PBS+IFA-treated mice) were measured after stimulation with SLP-8348 (10 μg/ml) for 5 days **(A)**. The same experiment was performed using SLP-8348, oxidized SLP-8348 (SLPOx-8348), and oxidation negative control (SLPB-8348) in the absence of adjuvant. IFN-γ levels in supernatants and proliferation index of CD4^+^ T cells (calculated as the ratio between the percentage of CFSE ^low^ CD4^+^ cells and CFSE^low^ CD4^+^ cells from PBS-treated mice) were measured after stimulation with SLP-8348 (10 μg/ml) for 5 days **(B)**. A representative figure of three independent experiments is shown (±SD, indicated by error bars). Asterisks indicate statistically significant differences by one-way ANOVA with Tukey's *post hoc* test (^*^p < 0.05).

In order to analyse SLP-8348 immunogenicity, one dose (10 μg/mouse) of SLP-8348 in combination with incomplete Freund's adjuvant (IFA) was subcutaneously injected into BALB/c mice and antigen specific T-cell proliferation in inguinal lymph nodes was evaluated 10 days post-injection. CD4^+^ T cells proliferation index as well as secretion of IFN-γ were significantly higher in the group of mice treated with SLP-8348/IFA respect to PBS/IFA-treated mice, after *ex vivo* stimulation with SLP-8348 (Figure 2A, Supplementary Figure 1). Surprisingly, when the experiments were performed in the absence of IFA, the group of mice treated with SLP-8348 alone exhibited similar responses as SLP-8348/IFA-treated mice, indicating that SLP-8348 is a highly immunogenic glycoprotein able to induce T cell proliferation and cytokine production in absence of adjuvant (Figure 2A, Supplementary Figure 1).

In order to establish whether the glycan structures present on SLP-8348 participate in the induction of a cell-mediated immune response, we evaluated the T-cell proliferation in inguinal lymph nodes from mice subcutaneously injected with SLP-8348, SLPOx-8348, or SLPB-8348. CD4^+^ T cell proliferation index as well as secretion of IFN-γ were significantly lower in the group of mice treated with SLPOx-8348 compared to SLP-8348-treated mice, after *ex vivo* stimulation with SLP-8348 (Figure 2B). The group of mice treated with SLPB-8348 displayed a similar response as SLP-8348-treated mice. These results indicate that SLP-8348 carbohydrates play an important role in the SLP-8348-induced T cell stimulation.

### Loss of Glycan Integrity Abrogates the Adjuvant Capacity of SLP-8348

**Figure 3:**
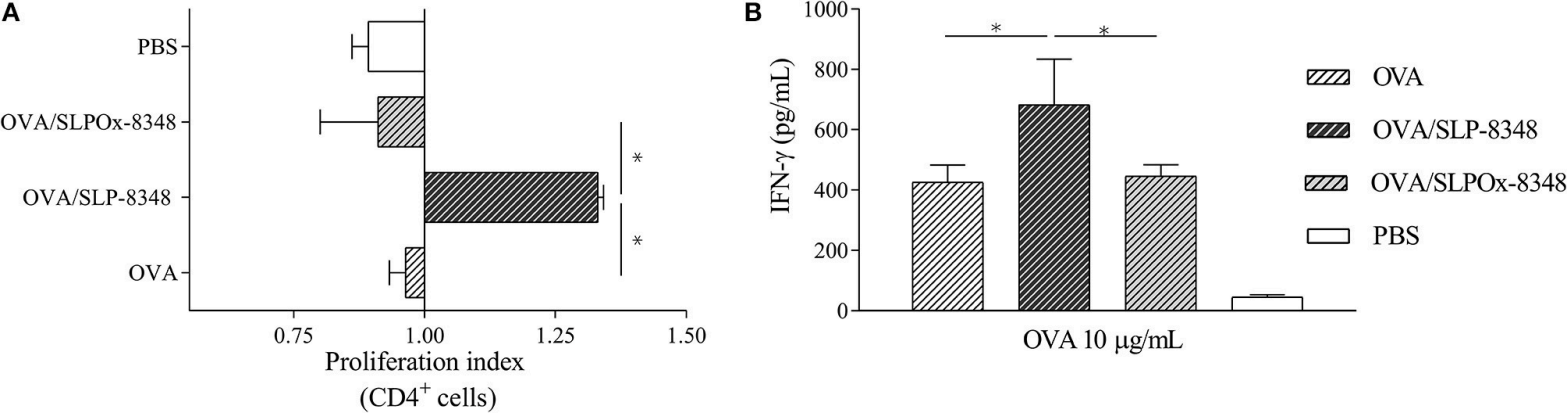
The adjuvant capacity of SLP-8348 is a glycan-mediated process. BALB/c mice were subcutaneously injected with one dose (10 μg/mouse) of SLP-8348 + OVA, oxidized SLP-8348 (SLPOx-8348) + OVA, PBS + OVA (OVA), or PBS (PBS). Cells were labeled with CFSE and stimulated *in vitro* with OVA (10 μg/ml) for 5 days. Proliferation index of CD4^+^ T cells in each experimental group was calculated as the ratio between the percentage of CFSE ^low^ CD4^+^ cells and CFSE^low^ CD4^+^ cells from OVA-treated mice **(A)**. IFN-γ levels in supernatants **(B)**. A representative figure of three independent experiments is shown (±SD, indicated by error bars). Asterisks indicate statistically significant differences by one-way ANOVA with Tukey's *post hoc* test (^*^p < 0.05).

Having shown that immunization with SLP-8348 can induce a specific cell-mediated immune response, and the crucial role of the carbohydrates of SLP-8348 in that effect, we subsequently explored the adjuvant potential of SLP-8348 using OVA as a model antigen. In order to evaluate the *in vivo* adjuvant capacity of SLP-8348, we injected subcutaneously OVA or OVA/SLP-8348 on BALB/c mice and after 10 days, cells from the inguinal lymph nodes were stimulated *in vitro* with OVA. Proliferation index of CD4^+^ T cells as well as secretion of IFN-γ against OVA were significantly higher in the group of mice treated with the combination of SLP-8348 and OVA compared to OVA-treated mice (Figures 3A,B). To determine if the carbohydrates from SLP-8348 participate in the enhanced response against OVA, we carried out the same experiments using the combination of SLPOx-8348 and OVA. The proliferation index of CD4^+^ T cells as well as secretion of IFN-γ against OVA were significantly lower in the group of mice treated with OVA/SLPOx-8348 compared to OVA/SLP-8348-treated mice (Figures 3A,B), indicating that oxidation of glycans abrogates the adjuvant capacity of SLP-8348. Levels of IFN-γ in absence of *in vitro* OVA stimulation are shown as Supplementary Figure 2.

### SLP-8348 Interacts With Different C-Type Lectin Receptors

**Figure 4:**
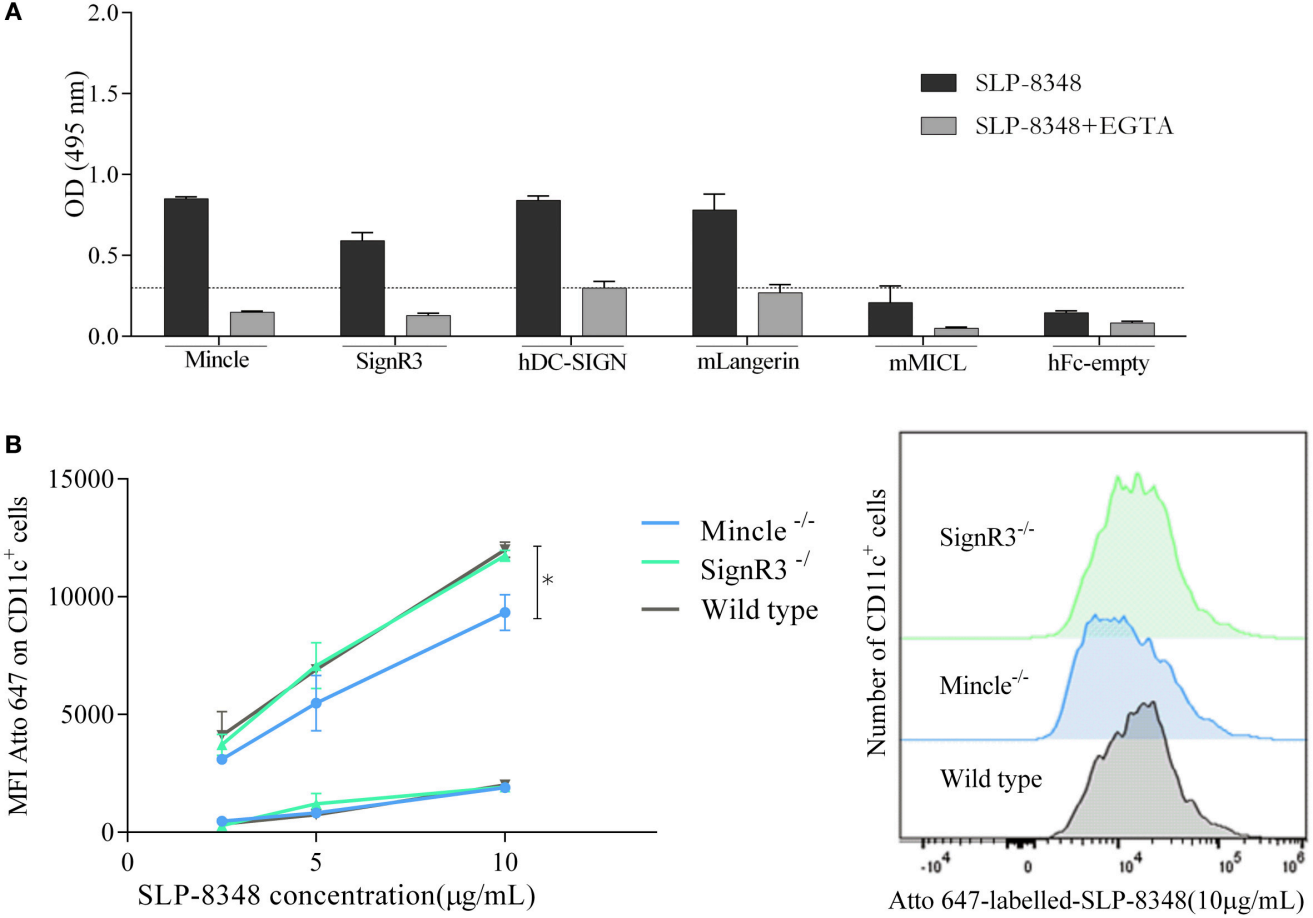
SLP-8348 is recognized and internalized by specific CLRs expressed on BMDCs. CLR-hFc fusion proteins were analyzed for the interaction with SLP-8348. Inhibition assays were performed using EGTA, a Ca^2+^ chelating agent **(A)**. Binding (4°C, squares) and internalization (37°C, circles) of Atto 647N-labeled-SLP-8348 at different concentrations on CD11c^+^ cells from C57BL/6 mice and CLR-deficient mice **(B)** A representative figure of three independent experiments is shown (±SD, indicated by error bars). Asterisks indicate statistically significant differences by one-way ANOVA with Tukey's *post hoc* test (^*^p < 0.05).

In order to identify possible CLRs that recognize SLP-8348, we performed an ELISA-based method using CLR–hFc fusion proteins (26). As shown in Figure 4A, glycoconjugates from SLP-8348 strongly reacted with Mincle, as well as with other receptors such as SignR3, hDC-SIGN and mLangerin. Taking into account that all the CLRs tested in this work recognized glycan structures in a Ca^2+^-dependent manner, we incubated the CLR-hFc fusion protein with SLP-8348 in the presence of EGTA. We observed that around 70% of the SLP-8348/CLR-hFc binding was abrogated with EGTA incubation. Additionally, a ~50% decrease was observed in the binding of SLPOx-8348 to the CLR-hFc fusion proteins compared to SLP-8348 (Supplementary Figure 3). All these findings confirm that the glycans present in SLP-8348 participate in the recognition by these CLRs in a Ca^2+^-dependent manner (Figure 4A).

After establishing the recognition pattern of CLRs on SLP-8348, we investigated whether this SLP-8348/CLRs engagement could induce internalization into BMDCs. To this end, Atto-647-labeled SLP-8348 was incubated with BMDCs from C57BL/6 wild type mice or CLR-deficient mice both at 4°C and 37°C and the fluorescence intensity was determined by flow cytometry. As shown in Figure 4B, SLP-8348 both interacted with BMDCs and was internalized by them. We found that SLP-8348 internalization was significantly reduced into BMDCs derived from Mincle^−/−^ mice but not from SignR3^−/−^ mice, indicating that the SLP-8348 uptake partially depends on glycan recognition by Mincle.

### SLP-8348 Induce Maturation of BMDCs Through Mincle Recognition

**Figure 5:**
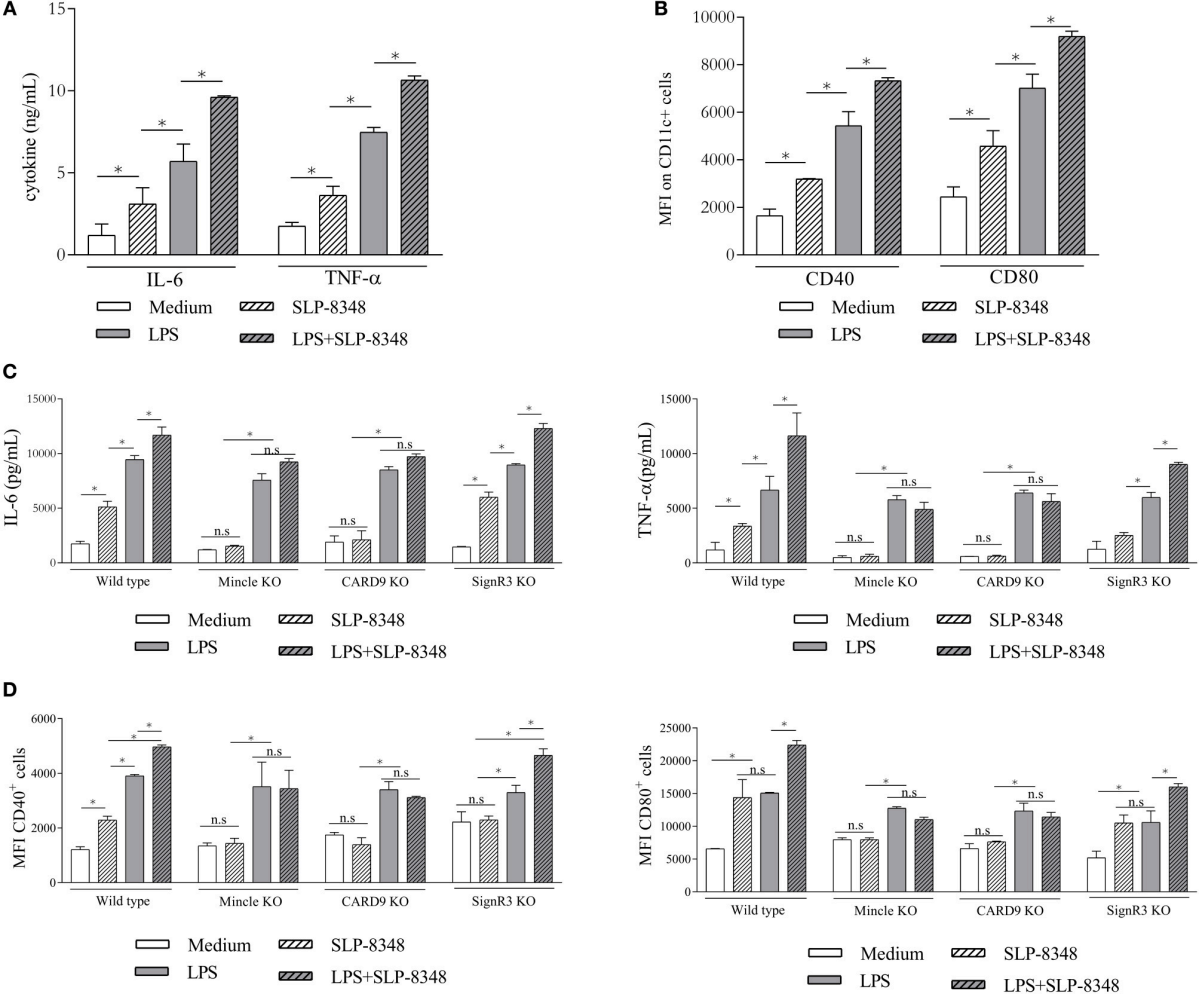
SLP-8348-induced activation of BMDCs is Mincle-dependent. BMDCs from C57BL/6 mice were stimulated with SLP-8348, LPS or the combination of both for 24 h. Culture supernatants and cells were collected and analyzed for the levels of IL-6 and TNF-α and for the cell surface expression of CD40 and CD80, respectively **(A,B)**. BMDCs from Mincle^−/−^ mice, CARD9^−/−^ and SignR3^−/−^ mice were stimulated with SLP-8348, LPS or the combination of both for 24 h. Culture supernatants were collected and analyzed by ELISA for IL-6 and TNF-α **(C)**. Cells were collected and cell surface expression of CD40 and CD86 on CD11c^+^ cells were analyzed by Flow cytometry **(D)** A representative figure of three independent experiments is shown (±SD, indicated by error bars). Asterisks indicate statistically significant differences by two-way ANOVA with Tukey's *post hoc* test (^*^p < 0.05); n.s: not significant; KO: knock-out.

To address the immunomodulatory properties of SLP-8348, we incubated SLPs with BMDCs from C57BL/6 wild type mice. After 24 h of incubation, SLP-8348 was able to induce increased levels of IL-6 and TNF-α, as well as the expression of CD40 and CD80 (Figures 5A,B). The same effect was observed using the combination of LPS and SLP-8348, generating higher levels of IL-6 and TNF-α, as well as higher expression of the costimulatory molecules CD40 and CD80 than LPS-stimulated BMDCs (Figures 5A,B).

Given the capacity of Mincle to recognize and internalize SLP-8348, we investigated whether this receptor or its adaptor protein CARD9 mediated the innate response to SLP-8348 in BMDCs. To this end, BMDCs derived from C57BL/6 wild type mice, Mincle-deficient mice, CARD9-deficient mice and SignR3-deficient mice were incubated overnight with SLP-8348, LPS or the combination of both. The absence of Mincle or CARD9 signaling on BMDCs resulted in a significantly reduced IL-6 and TNF-α production as well as CD40 and CD80 expression upon SLP-8384 stimulation compared to SignR3 or wild type BMDCs (Figures 5C,D). The same effect was observed using the combination of LPS and SLP-8348, demonstrating that Mincle and its signaling adaptor CARD9 contribute to the stimulation triggered by SLP-8348 and the modulation elicited by SLP-8348 on LPS-treated BMDCs (Figures 5C,D).

**Figure 6:**
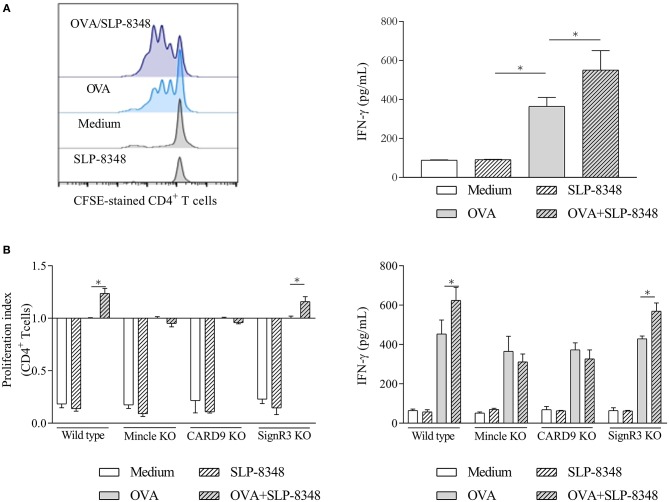
Interaction between SLP-8348 and Mincle favors the adjuvanticity of SLP-8348. BMDCs from C57BL/6 mice were stimulated with SLP-8348, OVA or the combination of both for 24 h. Cells were collected and exposed to CFSE-labeled CD4^+^ T cells from OT-II mice. Proliferation of CD4^+^ T cells (evaluated on CFSE-stained CD4^+^T cells) and IFN-γ levels in supernatants were measured after 5 days **(A)**. The same experiment was performed using BMDCs from CLR-deficient mice. Proliferation index of CD4^+^ T cells within each experimental group was calculated as the ratio between the percentage of CFSE ^low^ CD4^+^ cells and CFSE^low^ CD4^+^ cells from OVA-treated BMDCs **(B)** A representative figure of three independent experiments is shown (±SD, indicated by error bars). Asterisks indicate statistically significant differences by two-way ANOVA with Tukey's *post hoc* test (^*^p < 0.05).

To further investigate whether the enhanced activation elicited by SLP-8348 affected their T-cell stimulatory function, we incubated OVA-treated BMDCs from wild type, Mincle^−/−^, CARD9^−/−^, and SignR3^−/−^ mice in the presence or absence of SLP-8348 with purified T cells from OT-II mice. Our results showed that SLP-8348 could enhance the OVA-specific T cell proliferation, since the simultaneous stimulation with OVA and SLP-8348 of BMDCs generated a higher IFN-γ production as well as higher CD4^+^ T cell proliferation compared to OVA-treated BMDCs (76.2 vs. 57.0%, respectively) (Figure 6A). Furthermore, the proliferation index of CD4^+^ T cell as well as the IFN-γ levels were significantly reduced when CD4^+^ T cells from OT-II mice were incubated with OVA/SLP-8348-treated BMDCs derived from Mincle^−/−^ mice or CARD9^−/−^ mice, compared to the same treatment using BMDCs derived from wild type or SignR3^−/−^ mice (Figure 6B). These results clearly indicate that Mincle and CARD9 signaling mediate an enhanced OVA-specific response induced by SLP-8348 on BMDCs.

## Discussion

Studies of the biological role that carbohydrates play in bacterial glycoproteins have been increasing over time. In fact, the ability to modify proteins by adding carbohydrates was considered exclusive of eukaryotic cells until the appearance of studies on bacterial S-layer glycoproteins (27–29). The attention that these proteins have gained derives from their exceptional physicochemical properties rendering them a unique organizational structure with high application potential in different areas of modern nanobiotechnology. Taking into account that in our previous report we demonstrated that SLP-8348 could enhance the proinflammatory response on LPS-stimulated macrophages in a Ca^+2^-dependent manner (21), we first wanted to analyse the role of the carbohydrates in this process. In this respect, the chemical oxidation of terminal glycans has been probed as an adequate strategy trying to understand the role of glycans in the regulation of host immunity (24). When SLP-8348 was treated with meta-periodate and used to stimulate LPS-treated macrophages, the levels of secreted IL-6 as well as the expression of CD40 and CD80 decreased to those corresponding to the stimulation with LPS alone, indicating that the carbohydrates present on SLP-8348 modulate the TLR-induced maturation of macrophages.

In order to characterize more in detail the immunological properties of SLP-8348, we focused on the study of the antigen specific T-cell proliferation after a single subcutaneous inoculation of SLP-8348. Our results demonstrate the capacity of SLP-8348 to induce a high T-cell proliferation and the production of IFN-γ, either in the presence or absence of adjuvant. The immunogenicity of a *Lactobacilli* SLP was also demonstrated by Kajikawa and colleagues, who showed that after repeated high dose intragastric immunization with *L. acidophilus* NCFM, specific antibodies against SlpA were generated (30). To gain insight in the SLP-8348 glycan-mediated immune response *in vivo*, we took advantage of the chemical oxidation approach and performed the same experiments with SLPOx-8348. Our results clearly demonstrate that the immunogenicity of SLP-8348 is based on the carbohydrates linked to the glycoprotein, since the re-stimulation of inguinal lymph nodes derived-cells of SLPOx-8348-treated mice with purified SLP-8348 induced lower levels of IFN-γ and lower proliferation index of CD4^+^ T cells compared to SLP-8348-treated mice. This role of the glycan structures on the immune response elicited by bacterial glycoproteins was also demonstrated by Horn and colleagues, who showed that the immune stimulation elicited by mannosylated Apa glycoproteins of *Mycobacterium spp*. relies on the presence of the glycan component and also on the extent of glycosylation (31).

Vaccine adjuvants have traditionally been defined as materials that enhance the immune response to vaccine antigens (32). Beyond the mechanism of action, it appears that adjuvants activate innate immune responses to create a local immuno-competent environment at the injection site and draining lymph nodes, that is required to enhance adaptive immunity to the co-administered antigen (32, 33). In accordance with our results, SLP-8348 was able to generate a cell-mediated immune response in the draining lymph node after a subcutaneous injection. Therefore, we decided to evaluate the *in vivo* adjuvant capacity of SLP-8348 using OVA as model antigen. In OVA/SLP-8348-immunized mice, the expansion of OVA-specific T cells and the production of IFN-γ were significantly higher than in the group of mice immunized with OVA alone, showing that SLP-8348 could enhance the activation and differentiation of T cells. This adjuvant capacity of SLP-8348 was also dependent on the biological activity of the SLP-8348 glycans, since the co-administration of OVA and metaperiodate treated-SLP-8348 induced a similar adaptive cellular immune response as the administration of OVA alone. Even though previous studies have demonstrated the adjuvant capacity of SLPs from *Lactobacillus* strains (34), this is the first report showing the real participation of the glycan moieties since the loss of the integrity of terminal glycans completely abrogated the effect. Further studies will be needed in order to test the potential antigenicity of the SLP-8348 in this immunization protocol since it could affect its adjuvanticity.

The evaluation of the adjuvant mechanism of action is a crucial step in the development of effective human vaccines. In this respect, the important role of the glycans in the adjuvant capacity of SLP-8348 led us to study the CLRs that could recognize this glycoprotein. The glycan moieties expressed on SLP-8348 were strongly recognized by the macrophage inducible C-type lectin receptor (Mincle) and to a lesser extent by human DC-SIGN and its murine ortholog SignR3. It was previously reported that binding of SlpA from *L. acidophilus* NCFM to DC-SIGN on human BMDCs (18) or to murine SignR3 (35) is responsible for induction of immunoregulatory signals triggered by the SLP. Also, it was recently reported that Mincle is involved in both the recognition and the immune modulation elicited by the SLP from the oral pathogen *Tannerella forsythia* (36). Since the ELISA-based method used to detect the binding of SLP-8348 with CLR-Fc fusion proteins can lead to false-positive results, it requires confirmation by additional methods (26). Therefore, after establishing the recognition pattern of CLRs on SLP-8348 we investigated the biological role of the SLP-8348/CLRs engagement. Our results indicate that BMDCs could internalize SLP-8348 in a Mincle-dependent fashion, since the absence of this receptor on the cell surface of BMDCs reduced the uptake of SLP-8348. In contrast, although SignR3 recognize SLP-8348 glycans, this CLR does not mediate the internalization of the glycoprotein.

To elucidate the mechanism that could explain the adjuvant capacity of SLP-8348 we performed *in vitro* studies employing GM-CSF derived BMDCs which, according to the literature, represent a mixture of DCs, macrophages, and (few) granulocytes (37). SLP-8348 was able to induce a maturation of BMDCs from wild type mice, with production of IL-6, TNF-α and the up-regulation of the co-stimulatory molecules CD80 and CD40. When BMDCs were exposed to a simultaneous stimulation with SLP-8348 and LPS, we observed an increased response compared to LPS-stimulated cells. These results partially agree with our previous results, since SLP-8348 did not induce a macrophage activation using RAW 264.7 cells by itself but could enhance the response to stimulation with *E. coli* LPS (21), showing that the same stimulus could trigger different responses depending on the cell type, origin and polarization (38). We demonstrate that SLP-8348 induced maturation of BMDCs in a Mincle-dependent process, since the lack of this receptor generated a significant reduction of the levels of IL-6, TNF-α and co-stimulatory molecules. In addition, our results indicate that CARD9 is also involved in the proinflammatory response elicited by SLP-8348 on BMDCs. CARD9 is a caspase recruitment domain-containing signaling protein that is involved in the signaling pathway of ITAM-coupled receptors in myeloid cells, such as Mincle (39, 41). Previous studies have shown that the stimulation of macrophages with the mycobacterial glycolipid trehalose-6,6′-dimycolate (TDM), a well-known ligand of Mincle, leads to NF-κB activation via the FcRγ/Syk/CARD9 pathway and results in production of proinflammatory cytokines and chemokines such as TNF-α, CXCL2, CXCL1, and IL-6 (41, 42). Moreover, Schoenen et al have demonstrated that Mincle is necessary for the induction of adaptive immunity using a synthetic analog of TDM as an adjuvant (42). More recently, Shah et al. showed that a synthetic glycolipid (analog to the GL1 from *Lactobacillus plantarum*) is able to signal through Mincle using a NFAT-GFP reporter cell line (43). By carrying out OVA-specific T-cell proliferation assays using BMDCs from Mincle-deficient and CARD9-deficient mice, we demonstrate that Mincle and its signaling adaptor CARD9 mediate the adjuvant capacity of SLP-8348. CARD9 is involved in the signaling pathway of different CLRs (39). The phenocopy of Mincle^−/−^ and CARD9^−/−^ BMDCs strongly suggests that Mincle is a CLR that is involved in the immunomodulatory activity of SLP-8348. It has been shown that Mincle can interact with ligands of diverse chemical origin (43–46). In this regard, further studies are needed to determine the glycan structure of *L. kefiri* CIDCA 8348, in order to characterize the nature of the carbohydrate residues specifically recognized by Mincle.

In conclusion, we have demonstrated that SLP from *L. kefiri* CIDCA 8348 is able to enhance the OVA-specific immune response by triggering maturation of antigen presenting cells through the recognition of glycan moieties by Mincle. These results constitute a significant contribution to the understanding of the role of glycans in the immunomodulation elicited by bacterial SLPs and generate a promising opportunity in the search for new adjuvants derived from non-pathogenic microorganisms.

## Data Availability

The raw data supporting the conclusions of this manuscript will be made available by the authors, without undue reservation, to any qualified researcher.

## Ethics Statement

This study was carried out in accordance with the recommendations of the Institutional Animal Care and Use Committee of the Facultad de Ciencias Exactas, Universidad Nacional de La Plata. The protocol was approved by the same committee (Protocol 006-00-18).

## Author Contributions

MM performed the experiments, analyzed data, and prepared the manuscript. PC contributed with mice immunizations and flow cytometry experiments. MA participated in SLPs purification and mice immunizations. TF contributed with reagents, designed and analyzed the experiments involving SLPOx-8348, and revised the manuscript. BL and MS conceived the work, analyzed data, and revised the manuscript.

### Conflict of Interest Statement

The authors declare that the research was conducted in the absence of any commercial or financial relationships that could be construed as a potential conflict of interest.
