# Supplementary material for: S-Layer Glycoprotein From Lactobacillus kefiri Exerts Its Immunostimulatory Activity Through Glycan Recognition by Mincle
Source: Front Immunol. 2019 Jun 26;10:1422. doi: 10.3389/fimmu.2019.01422 (PMC6607945; doi:10.3389/fimmu.2019.01422)
Supplement: Supplementary file 1 [file Data_Sheet_1.docx]

**Supplementary Figure 1**


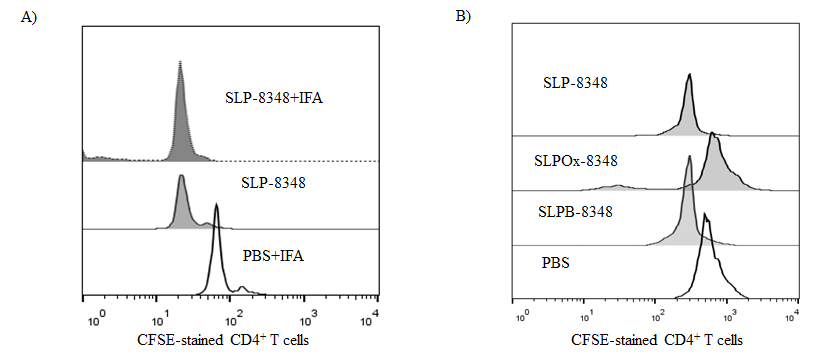


**Supplementary Figure 1.** Proliferation of CD4^+^ T cells (evaluated on CFSE-stained CD4^+^T cells) in cells from inguinal lymph nodes from immunized mice.

**Supplementary Figure 2**

**
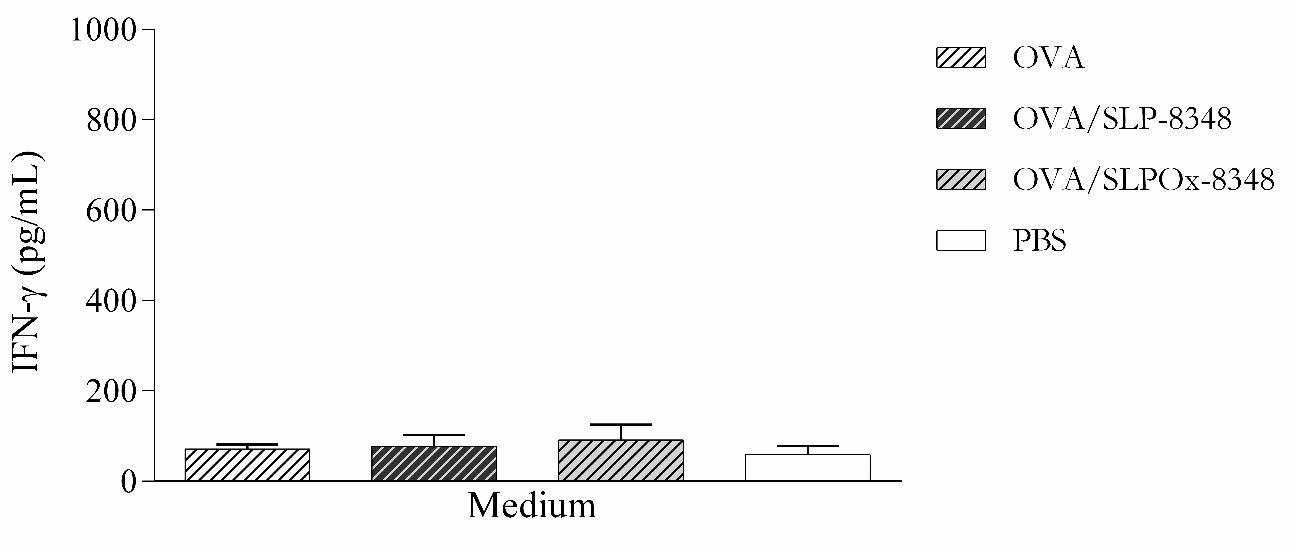
**

**Supplementary Figure 2.** IFN-γ levels in supernatants of cells from inguinal lymph nodes obtained from BALB/c mice subcutaneously injected with one dose (10 µg/mouse) of SLP-8348 + OVA, oxidized SLP-8348 (SLPOx-8348) + OVA, PBS + OVA (OVA), or PBS (PBS) in absence of in vitro antigenic stimulation.

**Supplementary Figure 3**


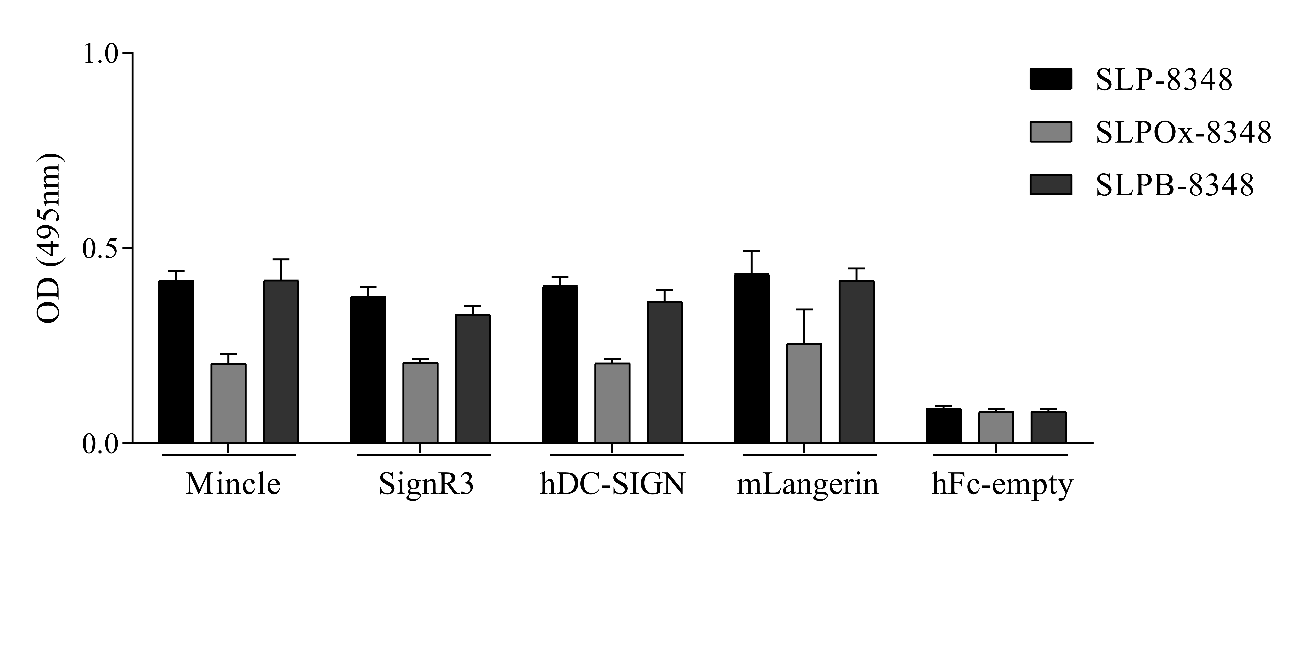


**Supplementary Figure 3.** CLR-hFc fusion proteins interaction with SLPOx-8348 and SLPB-8348.
